# Metabolic basis of post-infectious sequelae after Ebola virus disease

**DOI:** 10.1007/s11306-026-02544-z

**Published:** 2026-10-10

**Authors:** Anna C. Sanford, Nell G. Bond, Samuel Ficenec, Charlotte Osterman, Payton Farkas, Emily Engel, Bronwyn Gunn, Donald S. Grant, Robert Samuels, Kevin J. Zwezdaryk, John S. Schieffelin

**Affiliations:** 1 Department of Pediatrics, Tulane University School of Medicine, 1430 Tulane Ave, Box 8408, New Orleans, LA 70112 USA; 2 Tulane University, New Orleans, LA USA; 3 Washington State University, Pullman, WA USA; 4 Kenema Government Hospital, Kenema, Sierra Leone

## Abstract

**Introduction:**

Ebola virus disease (EVD) survivors often present with clinical sequelae after acute disease resolution, called post-Ebola syndrome (PES). Why some survivors develop these sequelae and others do not is poorly defined. Altered metabolism has been noted in acute EVD but has not been studied in PES. Here, we identified distinct metabolic pathways in EVD survivors with PES compared to asymptomatic survivors and potential biomarkers of PES.

**Objectives:**

The purpose of this study was to determine if EVD survivors with PES demonstrated differential metabolism compared to asymptomatic EVD survivors.

**Methods:**

Plasma was collected from EVD survivors in Sierra Leone as part of an ongoing cohort study. Untargeted metabolomics (LC-MS/MS) was performed on survivors with and without sequelae as well as household contacts. Univariate, multivariate, biomarker and pathway analyses were performed in MetaboAnalyst. Spearman’s R correlation was performed using JMP.

**Results:**

We identified differential expression of metabolites involved in multiple metabolic pathways in EVD survivors with PES compared to asymptomatic survivors. These pathways include the tricarboxylic acid cycle as well as amino acid, nucleotide, and short chain fatty acid metabolism. Several of these pathways are associated with immune dysfunction. The identified metabolites have potential use as biomarkers of PES.

**Conclusion:**

EVD survivors with PES exhibit differing metabolism compared to asymptomatic EVD survivors in several pathways. Other studies have linked these pathways to immune dysfunction and chronic viral infection, however further immunometabolic studies of EVD survivors with PES are needed.

**Supplementary Information:**

The online version contains supplementary material available at https://doi.org/10.1007/s11306-026-02544-z.

## Introduction

Many Ebola virus disease (EVD) survivors develop post-Ebola syndrome (PES) which consists of heterogenous sequelae including musculoskeletal, cardiopulmonary, neurologic, and constitutional (headache, fatigue) symptoms often identifiable long after acute illness (Etard et al., 2017, Sneller et al., 2019, Sanford et al., 2025). The mechanisms driving the development of these sequelae in some survivors but not others remain unclear. With improvement in the treatment of Ebola virus (EBOV) infected individuals, it is likely there will be an increase in survivors with PES. Therefore, a better understanding of the etiology of PES is crucial for identification of survivors at risk for PES and for future PES treatments.

Metabolomics can elucidate metabolic changes after viral infection and, therefore, suggest markers of post-viral sequelae for further investigation. Despite its utility, metabolomics has been used sparingly in studies of post-viral sequelae, although there has been work focusing on metabolic response in Long COVID (Wong et al., 2023) and HIV sequelae (Dickens et al., 2015). Ebola virus has been shown to alter fatty acid and amino acid metabolism in cells stimulated with virus-like particles (Tang et al., 2023), and EVD fatalities have been associated with a decrease in plasma free amino acids (Eisfeld et al., 2017). However, metabolic markers of PES have not been described. This raised the question of whether altered metabolism persists in EVD survivors with PES, as well as implications of identified metabolic changes. We hypothesized that EVD survivors with PES would demonstrate a unique metabolic profile compared to asymptomatic survivors and aimed to identify possible biomarkers of PES for further study.

## Methods

### Study design and population

A convenience sample of plasma was collected as part of an ongoing cohort study of EVD survivors and household contacts (HCs) in Sierra Leone as previously described (Bond et al., 2021). Survivors with musculoskeletal/gastrointestinal (MSK/GI; *n* = 18) or cardiopulmonary (CP; *n* = 19) sequalae were included in the PES group (*n* = 37). Samples were from three previously identified groups- EVD survivors with PES (MSK/GI or CP sequelae), asymptomatic glycoprotein (GP) IgG positive survivors (*n* = 20), and asymptomatic GP IgG negative HCs (*n* = 20). The HCs were selected to have a similar demographic background to survivors (age, sex, sample collection date) while being from the same region as EVD survivors. Some HCs were from the same households as survivors included in this study while others were from separate households. Samples were manually selected from a limited number of available plasma samples. We aimed to have a similar distribution of age, percent female, BMI and median sample collection date between groups. Logistic regression was performed to analyze differences in demographic characteristics between groups (Table 1). The primary analysis was between EVD survivors with PES and asymptomatic survivors (Supplementary Fig. 1). Secondary analyses included comparing EVD survivors with PES to household contacts and comparing sequelae groups in survivors with PES (musculoskeletal/gastrointestinal and cardiopulmonary).

### Clinical characteristics

Survivors in the MSK/GI and CP sequelae groups were identified from our previous studies (Bond et al., 2021, Ficenec et al., 2022). Sequelae considered MSK/GI sequelae include physical exam signs: decreased joint range of motion, extremity edema or effusion, joint tenderness to palpation, and abdominal tenderness to palpation. We prioritized including survivors with more frequent MSK/GI sequelae, although only one time point per survivor was analyzed in this study. Survivors who presented with multiple cardiopulmonary signs or symptoms were prioritized for inclusion in the CP group. Sequelae considered to be cardiopulmonary in origin included symptoms of chest pain, heart palpitations, shortness of breath, or a cough (Ficenec et al., 2022). Physical exam findings included as cardiopulmonary sequelae were the absence of S1 or S2 heart sounds, the presence of murmurs, rubs or gallops, an abnormal heart rate, an irregular heart rhythm, lower extremity edema, poor distal pulses, or the presence of wheezes, rales, rhonchi on auscultation or dullness to percussion of the posterior chest (Ficenec et al., 2022). Asymptomatic GP IgG+ survivors were defined as asymptomatic based on principal components analysis clustering as defined in our previous study (Bond et al., 2021). All survivors with MSK/GI sequelae were grouped into the MSK or MSK/GI cluster at first visit from our previous study. Most of the survivors with cardiopulmonary sequelae were grouped in the cardiac/GI cluster at first visit but three survivors did not cluster into a sequelae group at first visit and were later noted to have cardiopulmonary sequelae. Demographic characteristics and body mass index (BMI) were collected at first visit. If BMI was not collected at first visit, then data from the nearest follow-up visit was used. EBOV-targeted immunization such as rVSV-ZEBOV and therapies such as monoclonal antibodies or antivirals were not available to our study population.

### Sample and data collection

Plasma samples were collected from March 2016 to April 2019. Clinical data were collected as close to plasma collection date as possible (Supplementary Table 1). Blood samples were collected by venipuncture and plasma was processed according to standard methods. Using LC-MS/MS, untargeted metabolomics was completed for each sample by Gigantest Inc. (Baltimore, MD). Samples were stored at −80 °C before extraction. Samples were loaded onto a filter plate and extraction was performed using cold acetonitrile containing 1% formic acid. Positive pressure was applied to collect eluent which was utilized for data acquisition. Reversed-phase chromatographic separation was performed using Thermo Scientific Vanquish on a Thermo Scientific Orbitrap IQ-X using positive and negative ionization modes. Metabolites were identified by accurate-mass match within 5 ppm and/or fragmentation-pattern matching against the mzCloud library.

### Data analysis

Univariate, multivariate, biomarker and pathway analyses were performed in MetaboAnalyst 6.0 (Pang et al., 2024). Peak intensities were used for analysis. Missing values (*n* = 2762, 8.9%) were replaced with 1/5 the minimum positive values of their corresponding variables. Data was filtered using MetaboAnalyst 6.0 presets: variables were removed if they had a relative standard deviation (standard deviation/mean) greater than 25% and 25% of variables were removed based on interquartile range (IQR), selecting for near constant values (lower tail of IQR). Data was normalized to median and log transformed (base 10). Logistic regression determined whether demographic characteristics predicted group membership using R Studio version 4.4.2 (Boston, MA). Significantly different metabolites on univariate analysis were defined as FC > 2.0 or < 0.5 (equivalent to |log2FC| >1) and adjusted p-value (FDR via Benjamini-Hochberg method) <0.05 using Student’s t-test. Hierarchical clustering was performed on the identified significant metabolites using Euclidian distance and Ward clustering in MetaboAnalyst. Spearman’s R correlation was performed in JMP, Version 18.1.0. (SAS Institute Inc., Cary, NC, 1989–2025). Network analysis of significant correlations of tricarboxylic acid (TCA) cycle metabolites was performed in Cytoscape Version 3.10.3 (Shannon et al., 2003). Principal components analysis (PCA) and partial least squares discriminant analysis (PLS-DA) were performed in MetaboAnalyst. Variable importance of projection (VIP) scores were calculated from the top 15 PLS-DA metabolites in Components 1 and 2. Significant pathways included in pathway analysis were required to have an impact score > 0.2 and FDR < 0.05.

For biomarker analysis, PLS-DA and Random Forest (RF) models were trained with a discovery cohort of 39 randomly selected survivors (approximately 2/3 of survivors). Metabolomic data were filtered on feature frequency and IQR, and data was normalized and log transformed (base 10). Data was filtered, normalized and transformed separately in the discovery and validation cohorts. One hundred runs of Monte-Carlo cross validation (MCCV) were performed on the discovery and validation models. The validation cohort was unexposed to the discovery model prior to analysis. The validation cohort consisted of the remaining 18 randomly selected survivors. Each cohort was randomly assigned a proportional number of survivors with PES and asymptomatic survivors. The top 10 most important metabolites contributing to the discovery model were tested on the validation cohort. The 10-metabolite panel was fixed. During validation, the prespecified 10-metabolite panel was refit in the PLS-DA and Random Forest models within each MCCV iteration of the independent validation cohort. No additional features were evaluated or selected in the validation model. Receiver operating characteristic (ROC) curves were generated from 100 cross validated runs for both discovery and validation cohorts. The reported 95% confidence intervals were derived from the distribution of AUC estimates across MCCV runs. These confidence intervals utilize the lower and upper 2.5th percentiles. Data visualization was performed in MetaboAnalyst, JMP and GraphPad Prism version 10.6.1 (Boston, MA).

### IRB determination and written consent

This study was approved by the Tulane University Institutional Review Board (IRB approval number: 701226) and the Sierra Leone Ethics and Scientific Review Committee (021/11/2024). Written informed consent was provided by adult participants ≥ 18 years, consent by a parent or guardian and assent in children 12–17 years, and consent by a parent or guardian in children < 12 years. Study personnel were all trained in ethics and research compliance.

## Results

### EVD survivors with PES display a unique metabolome

**Fig. 1:**
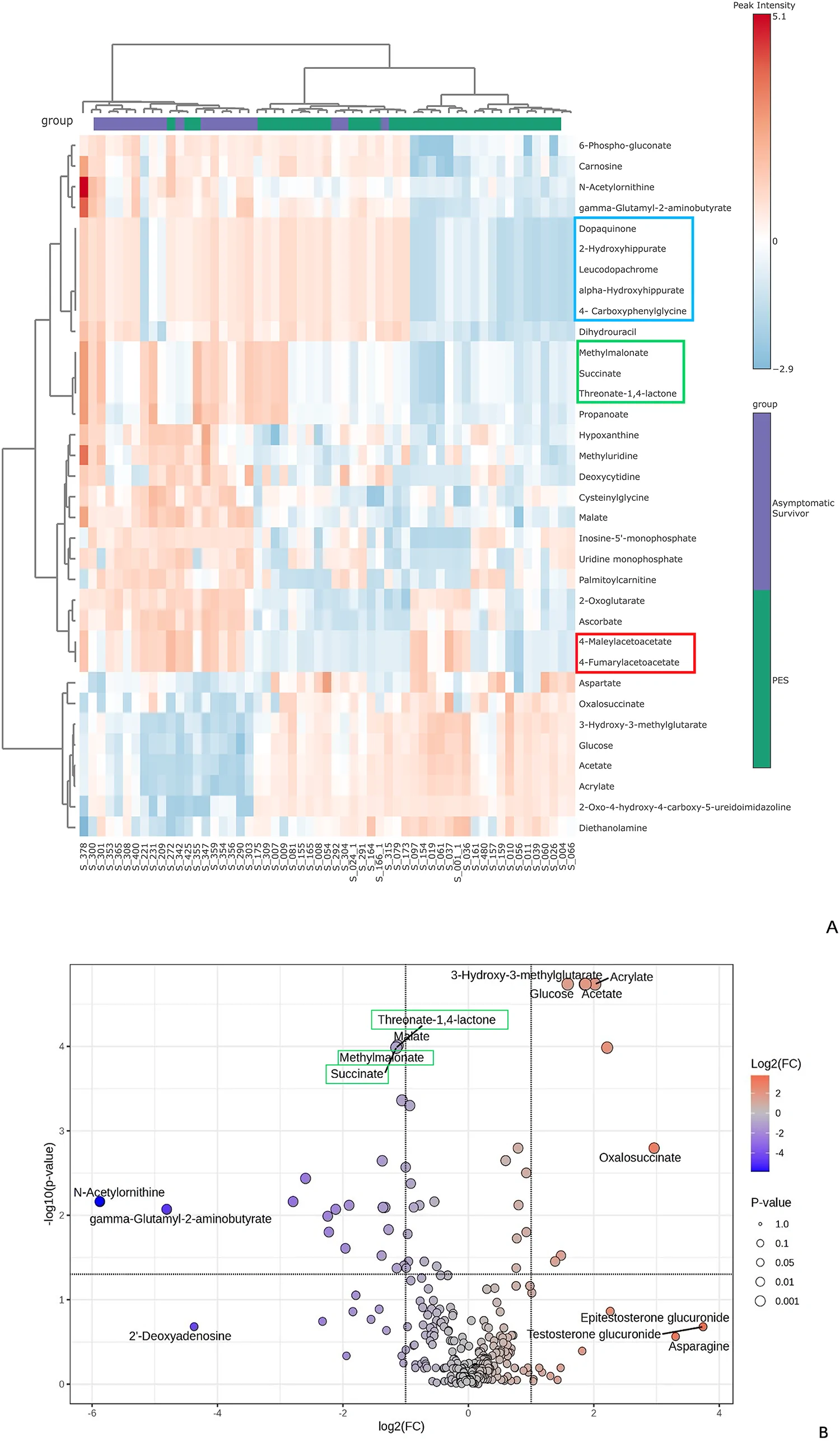
EVD Survivors with PES demonstrate a unique metabolic profile compared to asymptomatic survivors. **A**: 26 metabolites are significantly downregulated in the PES group and 8 metabolites are significantly upregulated compared to asymptomatic survivors. Differential metabolites are defined as FC > 2.0 or < 0.5 (equivalent to |log2FC| >1) and adjusted p-value (FDR) <0.05. Peak intensities were normalized to median and log10 transformed. Analysis and visualization performed in MetaboAnalyst. Peak intensities consistent with multiple metabolites are outlined in the same color. **B**: Volcano plot demonstrates differentially expressed metabolites in EVD survivors with PES compared to asymptomatic survivors. Metabolites to the right of X = 0 on the X-axis represent upregulated metabolites in the PES group compared to asymptomatic survivors, while metabolites to the left are downregulated in the PES group compared to asymptomatic survivors. The Y-axis demonstrates significance, with *p*<.05 corresponding to a -log10 value of 1.3, Metabolites above this line were significantly different between survivors with and without PES. P-value displayed is adjusted p-value (FDR). The size of the metabolite marker indicates significance, as demonstrated to the right of the figure. Analysis and visualization performed in MetaboAnalyst.* FC*fold change

EVD survivors with PES (*n* = 37) demonstrated an altered metabolic profile compared to asymptomatic EVD survivors (*n* = 20), characterized largely by downregulation of multiple metabolic pathways (Fig. 1A; adjusted p-value for all comparisons: <0.05). Age, sex, BMI, and sample collection date were not significant for predicting PES when comparing EVD survivors with PES and asymptomatic survivors (Table 1). There were no significantly different metabolites when comparing survivors with MSK/GI sequelae (*n* = 18) to survivors with cardiopulmonary sequelae (*n* = 19). There were no significantly different metabolites when comparing survivors with PES and asymptomatic HCs (*n* = 20).

**Table 1:** Logistic regression included age, sex, BMI and sample collection date to predict group membership

	PES (*n* = 37)	Asymptomatic survivors (*n* = 20)	*p*-value	PES & asymptomatic survivor (*n* = 57)
Median age (y)	30	28	0.22	29
Female (%)	59.5	60	0.99	59.6
Median BMI (kg/m^2^)	24.5	22.1	0.11	23.1
Median sample collection date	2017-03-08	2017-11-26	0.11	2017-11-21
	PES (*n* = 37)	Household Controls (*n* = 20)	p-value	PES & Household Controls (*n* = 57)
Median age (y)	30	29.5	0.75	30
Female (%)	59.5	60.0	0.98	59.6
Median BMI (kg/m^2^)	24.5	23.5	0.49	24.4
Median sample collection date	2017-03-08	2017-07-14	0.50	2017-04-24
	MSK/GI (*n* = 18)	CP (*n* = 19)	p-value	MSK/GI & CP (*n* = 37)
Median age (y)	30	25	0.82	30
Female (%)	66.7	52.6	0.89	59.5
Median BMI (kg/m^2^)	27.2	22.7	0.11	24.5
Median sample collection date	2017-02-12	2017-04-24	0.02*	2017-03-08

No characteristic was significant in predicting PES when comparing EVD survivors with PES and asymptomatic survivors or EVD survivors with PES and household controls. EVD survivors with cardiopulmonary (CP) sequelae had a significantly later collection date compared to survivors with musculoskeletal or gastrointestinal (MSK/GI) sequelae. Logistic regression performed in R Studio. *PES* Post-Ebola syndrome, *EVD* Ebola virus disease

Using univariate analysis, five hundred and forty-four metabolites were identified and 34 were differentially expressed when comparing EVD survivors with PES and asymptomatic survivors. Peak intensities consistent with twenty-six metabolites were significantly decreased and eight metabolites were significantly increased in this comparison (Fig. 1A, B; Supplementary Table 2). Several metabolites demonstrated shared analytical features and could not be further differentiated with untargeted analysis (Supplementary Table 2). Key pathways demonstrating altered metabolism in EVD survivors with PES compared to asymptomatic survivors included nucleotide metabolism, the TCA cycle, and short chain fatty acid (SCFA) metabolism.

**Fig. 2:**
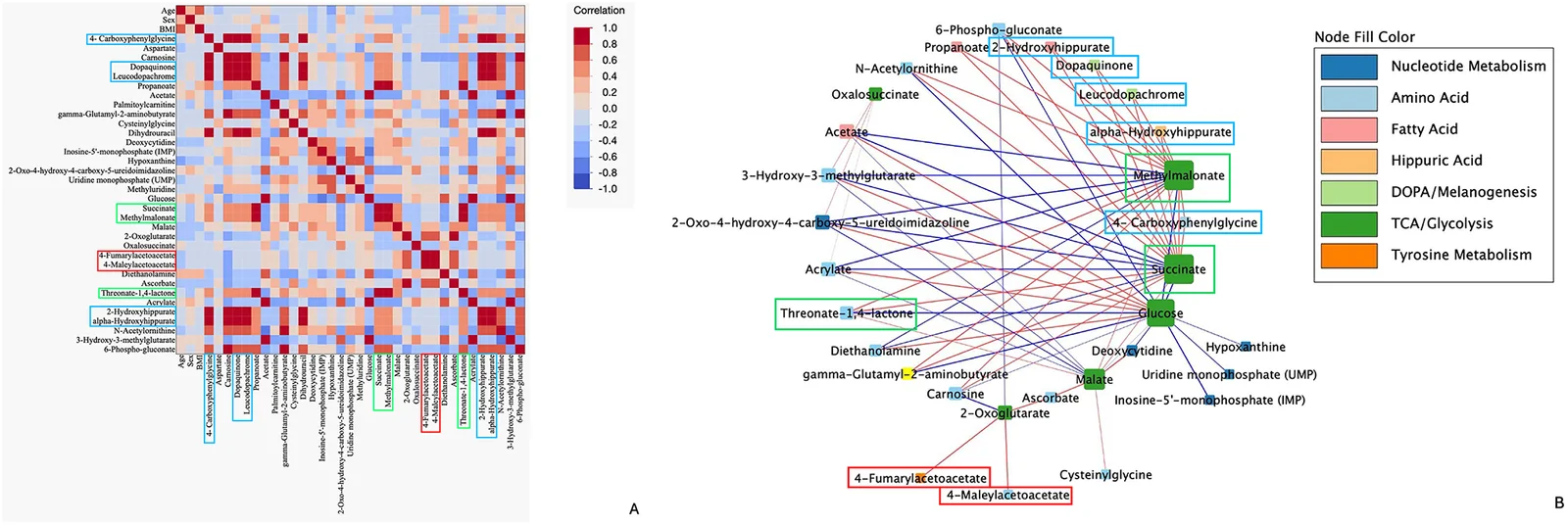
Correlation analysis reveals significant interactions between metabolic pathways in EVD survivors with PES. **A**: Heatmap of Spearman’s rank correlation analysis of EVD survivors with PES including demographic characteristics (age, sex, BMI) and significantly differential metabolites. Included metabolites were considered significant if |log2FC| was > 1.0 and FDR < 0.05 when comparing EVD survivors with PES to asymptomatic survivors. Correlations between variables of survivors with PES are red if positive and blue if negative. **B**: Network analysis of TCA cycle metabolites in EVD survivors with PES. Node size was determined by the number of connections for the metabolite (larger node corresponds to more connections). Node fill color demonstrates which pathway the metabolite belongs to. Line thickness was determined by the p-value of the correlation (smaller p-value corresponds to thicker line). Line color demonstrates whether association is positive (red) or negative (blue). Relationships were determined by Spearman’s rank correlation performed in JMP. Only significant relationships (FDR < 0.05) were included. Correlation network visualized using Cytoscape. Supplementary Table 3 contains rho values. Peak intensities consistent with multiple metabolites are outlined in the same color

Spearman’s R correlation analysis of EVD survivors with PES demonstrated significant relationships of metabolites in the TCA cycle, amino acid, fatty acid, and nucleotide metabolism pathways (Fig. 2A; Supplementary Table 3). There was no significant correlation between demographic characteristics (BMI, age, sex) or any metabolite in the PES group; however, BMI significantly correlated with age (positive correlation). Given the number of TCA metabolites affected, we further illustrated this pathway via network analysis (Fig. 2B).

**Fig. 3:**
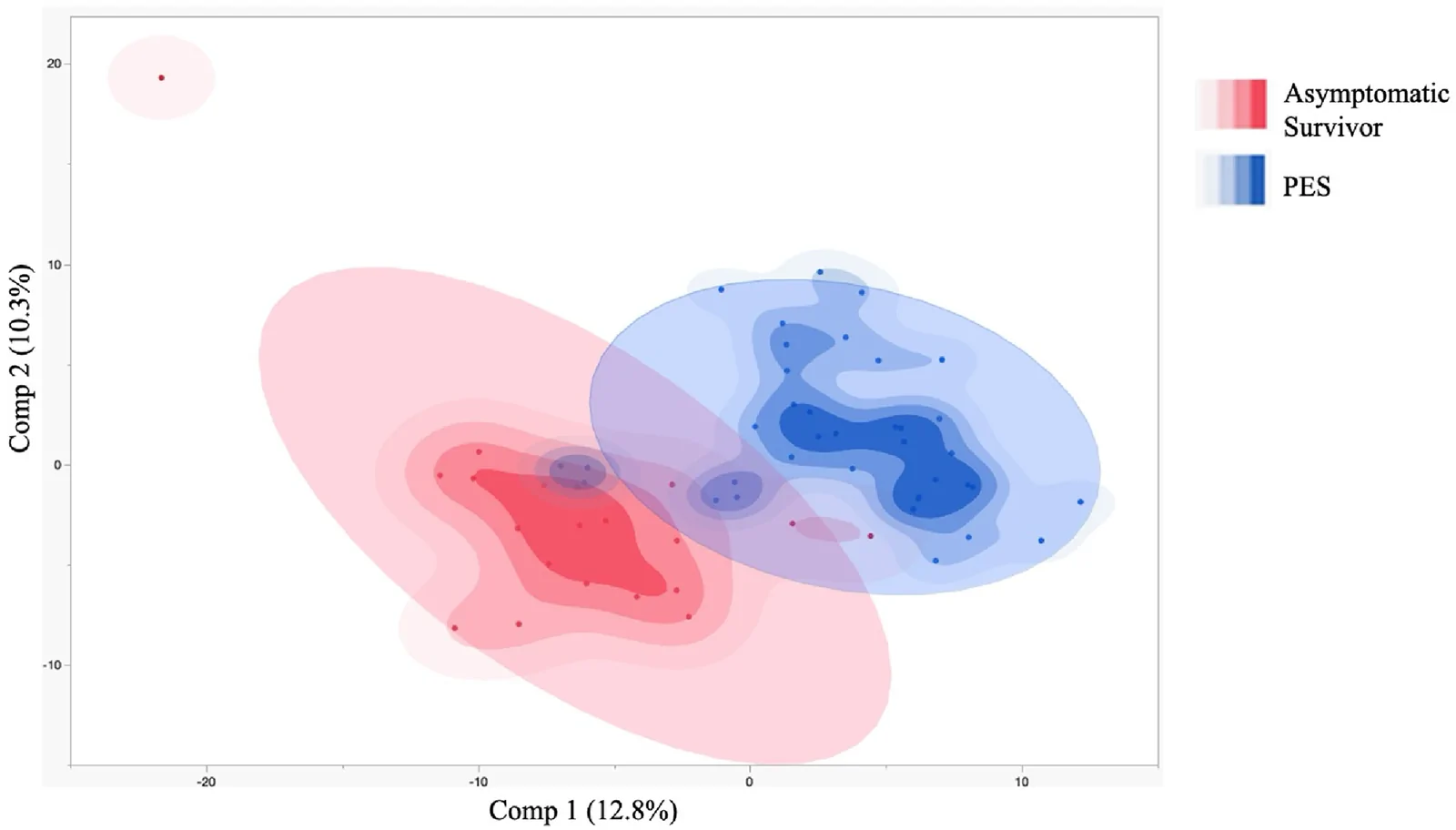
EVD survivors with PES and asymptomatic survivors separate into distinct groups based on metabolic profile. Partial least squares discriminant analysis (PLS-DA) demonstrates separation of PES and asymptomatic survivor groups. The highest accuracy PLS-DA model included 2 components with an accuracy of 0.86, R2 = 0.66, and Q2 = 0.45. Data analyzed in MetaboAnalyst and visualized in JMP.

PCA and PLS-DA showed separation of EVD survivors with PES and asymptomatic survivors based on metabolic profile (Supplementary Fig. 2, Fig. 3). Metabolites associated with the TCA cycle, SCFA metabolism, and glucose all demonstrated importance for Components 1 and 2 of the PLS-DA model (Supplementary Table 4).

### Survivors with PES demonstrate downregulation of multiple metabolic pathways

**Fig. 4:**
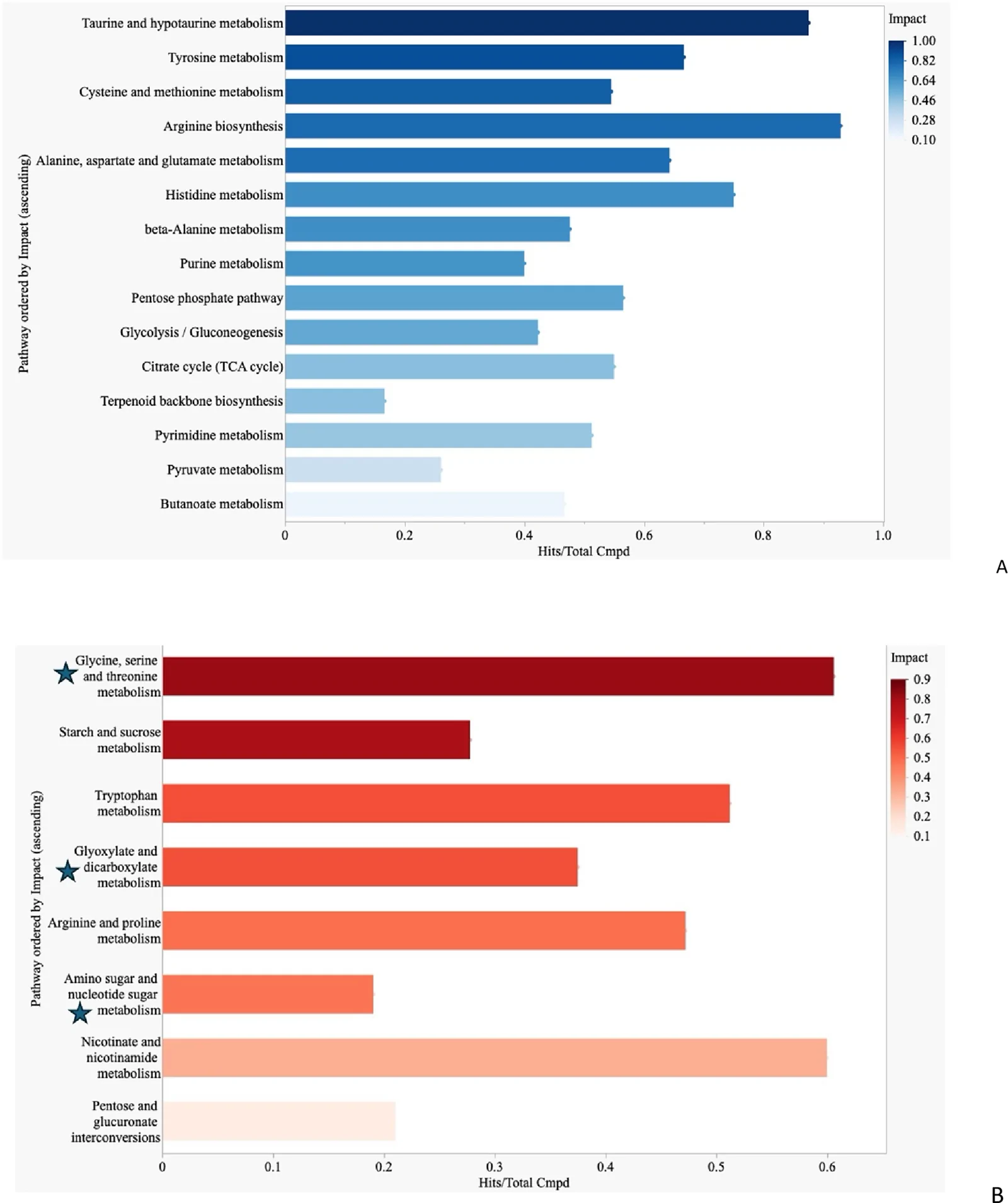
EVD survivors with PES demonstrate significant differences in metabolic pathways. Pathway analysis demonstrates significantly impactful pathways in EVD survivors with PES compared to asymptomatic survivors. **A** demonstrated pathways with primarily downregulated metabolism and **B** demonstrated pathways with significantly upregulated or “tie” pathways in PES compared to asymptomatic survivors. Pathways with an equal number of upregulated and downregulated (“tie” pathways) metabolites are denoted by a star in **B**. Pathways ordered by impact score. Impact score required to be > 0.2 and FDR < 0.05 for inclusion. Pathway analysis performed in MetaboAnalyst and data visualized using JMP

Pathway analysis demonstrated significantly different metabolic pathways in EVD survivors with PES compared to asymptomatic survivors (Fig. 4A, FDR < 0.05). The most impactful downregulated pathways involved amino acid metabolism (taurine, hypotaurine, tyrosine, cysteine and methionine, among others). Nucleotide, TCA cycle, glycolysis, and pyruvate metabolism pathways were also significantly downregulated. Fewer pathways were significantly upregulated or were significantly involved with an equal number of up and downregulated metabolites (Fig. 4B). Significantly upregulated pathways included starch and sucrose metabolism, tryptophan metabolism, arginine and proline metabolism, nicotinate and nicotinamide metabolism, pentose and glucuronate interconversions.

### Biomarker analysis: metabolites predict Post-Ebola syndrome

**Fig. 5:**
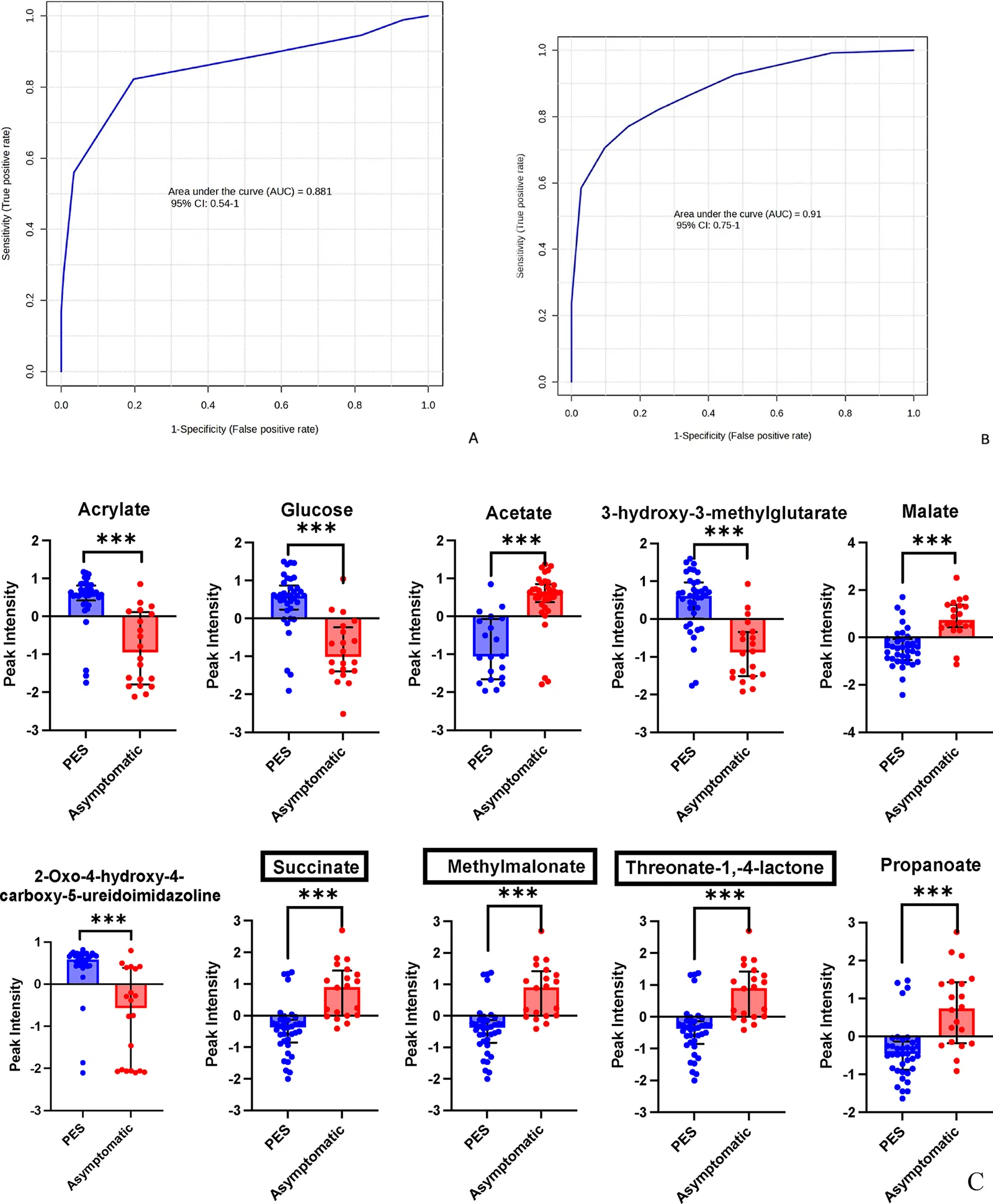
Receiver operating characteristics (ROC) curves demonstrate ability of PLS-DA and RF models to distinguish between Ebola survivors with PES and asymptomatic survivors. The top ten most important metabolites in both models were acrylate, glucose, acetate, 3-hydroxy-3-methylglutarate, malate, 2-Oxo-4-hydroxy-4-carboxy-5-ureidoimidazoline, succinate, methylmalonate, threonate-1,−4-lactone and propanoate. Data analyzed and visualized in MetaboAnalyst. Discovery models available as Supplementary Fig. 3A, B. **A**: PLS-DA validation model. **B**: Random Forests (RF) validation model. **C**: Boxplots of individual model features. Significant metabolites defined via univariate analysis as previously described (|log2FC| >1 with FDR < 0.05). Boxplots demonstrate median with IQR.*** = adjusted p-value (FDR) <0.001. Metabolites with shared analytical features are outlined

Biomarker analysis identified ten metabolites predictive of PES when comparing EVD survivors with PES to asymptomatic survivors. The ten metabolites predictive of PES were the same in the RF and PLS-DA models, both with areas under the curve > 0.8 (Supplementary Fig. 3A, B). These metabolites included SCFA and TCA cycle metabolites, among others. These ten metabolites were also significant in the univariate analysis comparing EVD survivors with PES and asymptomatic survivors (Figs. 1A and 5C). These predictive metabolites were then used in a separate validation cohort to predict PES and again demonstrated areas under the curve > 0.8 (Fig. 5A, B). Predictive accuracy for the validation cohort was 0.799 and 0.796 for the for the PLS-DA and RF models, respectively.

## Discussion

Here, we demonstrate that Ebola survivors with PES, compared to asymptomatic survivors, exhibit downregulated metabolism of multiple metabolic pathways. Downregulation was observed in the TCA cycle, amino acid and nucleotide metabolism pathways. SCFAs (propanoate, acetate) were also significantly altered. Metabolites involved in these pathways were identified as potential biomarkers using two additional models: RF and PLS-DA. The same ten metabolites were identified via both analyses, reinforcing the possibility that these metabolites play a role in PES.

Our findings are consistent with metabolic changes seen in cells stimulated with EBOV-like particles (Tang et al., 2023) as well as chronic viral infections (Tarancon-Diez et al., 2019, Saeidi et al., 2018). As previously discussed, amino acid metabolism is affected in cells stimulated with EBOV-like particles (Tang et al., 2023). In people living with human immunodeficiency virus (HIV), changes to amino acid metabolism and signs of mitochondrial disruption including TCA cycle dysfunction have been noted in transient controllers of HIV compared to persistent controllers (Tarancon-Diez et al., 2019). Altered mitochondrial function has been associated with T-cell exhaustion in chronic infections such as HIV, hepatitis B virus (HBV), and lymphocytic choriomeningitis virus (LCMV) (Saeidi et al., 2018). Our findings could indicate similar immune changes in EVD survivors with PES. While our results are from plasma samples and cannot be directly linked to the cellular level, these findings warrant further investigation.

The changes seen in SCFA among survivors with PES (increased acetate, decreased propanoate) could be due to dysregulation of the gut microbiome in EVD survivors with PES. Reduced SCFA-producing symbionts have been noted in persons with Long COVID (Zhang et al., 2023). SCFAs can influence immune response, typically encouraging an anti-inflammatory response. SCFAs have also been shown to modulate CD8 + T-cell activity (Nastasi et al., 2017).

Although several studies have focused on immune biomarkers of PES (LaVergne et al., 2020, Wiedemann et al., 2020), no clear profile has been identified. Prior research in CD8 + T-cell activity in PES has demonstrated that more survivors with PES demonstrate an EBOV-specific CD8 + T-cell response compared to asymptomatic survivors (LaVergne et al., 2020). As immune function and differentiation are mediated by metabolic pathways, we questioned if Ebola virus disrupts metabolic homeostasis. We observed changes in the TCA cycle that are associated with decreased CD8 + T-cell activity in chronic infections or post-viral syndromes (Saeidi et al., 2018). Additionally, SCFA changes have been associated with other post-viral sequelae (Zhang et al., 2023) and influence CD8 + T-cell activity (Nastasi et al., 2017).

There are several limitations to our study. First, the sample size is a limitation. It is possible that additional key metabolites associated with PES would be identified in a larger study. Corrections for multiple comparisons were made for metabolomics analyses to minimize possibility of type one error (described in methods). This could make identifying significance more challenging; however, these corrections are necessary for stringent analysis of metabolomics data. Due to the small sample size, we chose to focus on two specific sequelae groups (musculoskeletal/gastrointestinal and cardiopulmonary). This could limit generalizability to the broader array of PES sequelae. A larger study which includes comparison of more sequelae groups would be an important future direction. Additionally, there was no significant difference in univariate analysis when comparing EVD survivors with PES and household contacts. This could be due to several reasons, including limited sample size, as trends for several metabolites were similar in this comparison but not statistically significant (data not shown). Another possibility is that EVD survivors without PES demonstrate unique metabolic changes which contribute to the significant difference between EVD survivors with and without PES. Furthermore, concurrent laboratory data was not available to compare to our metabolomic data (i.e. biochemistry tests, metabolic parameters, inflammatory markers). This would have been useful data to correlate with our metabolomics results. Due to the nature of the outbreak, we also do not have clinical or laboratory data from the acute phase of EVD, including metabolomic data or data on any treatments provided during acute illness. We also lack longitudinal samples. This data would be important for distinguishing persistent metabolic changes from acute EVD from alterations specific to PES as well as metabolic changes that may be associated with treatment. Another limitation was the time between plasma sample collection and clinical assessment for some survivors (Supplementary Table 1). Gaps between clinical assessment and plasma sample collection occurred with some survivors, however we prioritized including survivors with clinical data and a plasma sample as close together as possible. Additionally, shared analytical features among some metabolites limits precision when describing those metabolites and their significance. Future targeted metabolomics focusing on these metabolites would be a beneficial next step. As we only studied plasma samples, we can only suggest potential cellular mechanisms of metabolic changes in EVD survivors with PES. Further studies at the cellular level, particularly looking at T-cell function and immunometabolism, are required to determine if the metabolic changes we observed are related to immune dysfunction.

In conclusion, EVD survivors demonstrate altered metabolism in the TCA cycle, amino acid, nucleotide, and SCFA pathways. Several of these pathways have been linked to immune dysfunction (particularly in CD8 + T cells) and chronic infection in studies of other viral infections. With our current data, any suggestion of viral persistence can only be hypothetical. Further studies of cellular immunometabolism in EVD survivors with PES are needed. Additionally, direct metabolomic comparison with a control group of post-viral sequelae after another viral infection (i.e. SARS-CoV-2) would help determine clinical relevance of our findings. Our plans for future work include more mechanistic studies to determine how the observed metabolic changes occur and how they relate to immune function. While our work focuses on metabolic changes in a specific group, EVD survivors with PES, we hope our work also demonstrates the utility of metabolomic study of post-viral sequelae more broadly.

## Supplementary Information

Below is the link to the electronic supplementary material.

Supplementary Material 1

Supplementary Material 2

## Data Availability

The metabolomics and metadata reported in this paper are available via Figshare (https://figshare.com/s/f0ddc51a4317441602a6, https://figshare.com/s/d3cd6da06139166ae9ec).
