# Supplementary material for: Metabolic basis of post-infectious sequelae after Ebola virus disease
Source: Metabolomics. 2026 Oct 10;22(6):170. doi: 10.1007/s11306-026-02544-z (PMC13653831; doi:10.1007/s11306-026-02544-z)
Supplement: Supplementary file 2 [file 11306_2026_2544_MOESM2_ESM.docx]

**Supplementary Data**

**Supplementary Tables**

| **Sample Collection Date (MM-YYYY)** | **Clinical Sequelae Date**  **(MM-YYYY)** | **Sequelae** |
| --- | --- | --- |
| 03-2016 | 03-2016 | MSK/GI |
| 02-2017 | 03-2016 | MSK/GI |
| 03-2016 | 03-2016 | MSK/GI |
| 03-2016 | 03-2016 | MSK/GI |
| 03-2016 | 03-2016 | CP |
| 02-2017 | 03-2016 | MSK/GI |
| 08-2018 | 03-2022 | CP |
| 04-2019 | 04-2019 | CP |
| 03-2016 | 03-2016 | MSK/GI |
| 02-2017 | 02-2017 | MSK/GI |
| 04-2019 | 04-2019 | CP |
| 04-2019 | 03-2020 | CP |
| 08-2018 | 03-2022 | CP |
| 03-2016 | 03-2016 | MSK/GI |
| 03-2017 | 03-2017 | MSK/GI |
| 03-2017 | 03-2017 | CP |
| 04-2019 | 04-2019 | CP |
| 03-2017 | 03-2017 | MSK/GI |
| 09-2016 | 09-2016 | MSK/GI |
| 03-2016 | 03-2016 | MSK/GI |
| 04-2019 | 03-2020 | CP |
| 04-2019 | 03-2020 | CP |
| 10-2016 | 04-2017 | CP |
| 04-2017 | 04-2017 | CP |
| 04-2017 | 04-2017 | CP |
| 04-2017 | 04-2017 | CP |
| 04-2016 | 04-2016 | CP |
| 04-2016 | 04-2017 | CP |
| 04-2016 | 04-2016 | MSK/GI |
| 03-2016 | 08-2016 | CP |
| 08-2016 | 08-2016 | CP |
| 07-2017 | 07-2017 | MSK/GI |
| 11-2017 | 11-2017 | MSK/GI |
| 11-2017 | 11-2017 | MSK/GI |
| 12-2017 | 11-2017 | MSK/GI |
| 12-2017 | 11-2017 | MSK/GI |
| 09-2018 | 03-2020 | CP |

Supplementary Table 1: Dates of plasma collection and sequelae for all survivors with sequelae. ID not shown to protect patient privacy.

|  | FC | log2(FC) | p.adjusted | -log10(p) |
| --- | --- | --- | --- | --- |
| Acrylate | 4.0574 | 2.0206 | 1.83E-05 | 4.7381 |
| Acetate | 3.6465 | 1.8665 | 1.83E-05 | 4.7381 |
| 3-Hydroxy-3-methylglutarate | 3.6228 | 1.8571 | 1.83E-05 | 4.7381 |
| Glucose | 2.9963 | 1.5832 | 1.83E-05 | 4.7381 |
| Malate | 0.45773 | -1.1274 | 9.78E-05 | 4.0099 |
| 2-Oxo-4-hydroxy-4-carboxy-5-ureidoimidazoline | 4.6347 | 2.2125 | 0.00010336 | 3.9857 |
| **Succinate** | 0.45074 | -1.1496 | 0.00010336 | 3.9857 |
| **Methylmalonate** | 0.45074 | -1.1496 | 0.00010336 | 3.9857 |
| **Threonate-1,4-lactone** | 0.45074 | -1.1496 | 0.00010336 | 3.9857 |
| Propanoate | 0.48027 | -1.0581 | 0.00043485 | 3.3617 |
| Oxalosuccinate | 7.7896 | 2.9615 | 0.0016024 | 2.7952 |
| Palmitoylcarnitine | 0.38619 | -1.3726 | 0.0022608 | 2.6457 |
| Ascorbate | 0.16542 | -2.5958 | 0.0036576 | 2.4368 |
| N-Acetylornithine | 0.017035 | -5.8753 | 0.0068859 | 2.162 |
| Deoxycytidine | 0.14416 | -2.7942 | 0.0068859 | 2.162 |
| Hypoxanthine | 0.26767 | -1.9015 | 0.0076167 | 2.1182 |
| **2-Hydroxyhippurate** | 0.38912 | -1.3617 | 0.0080669 | 2.0933 |
| **Dopaquinone** | 0.38912 | -1.3617 | 0.0080669 | 2.0933 |
| **Leucodopachrome** | 0.38912 | -1.3617 | 0.0080669 | 2.0933 |
| **alpha-Hydroxyhippurate** | 0.38912 | -1.3617 | 0.0080669 | 2.0933 |
| **4- Carboxyphenylglycine** | 0.38912 | -1.3617 | 0.0080669 | 2.0933 |
| Dihydrouracil | 0.39851 | -1.3273 | 0.0080669 | 2.0933 |
| gamma-Glutamyl-2-aminobutyrate | 0.035676 | -4.8089 | 0.0084969 | 2.0707 |
| Cysteinylglycine | 0.23083 | -2.1151 | 0.0084969 | 2.0707 |
| 2-Oxoglutarate | 0.21137 | -2.2421 | 0.010247 | 1.9894 |
| Inosine-5'-monophosphate (IMP) | 0.4141 | -1.2719 | 0.014766 | 1.8307 |
| 6-Phospho-gluconate | 0.21403 | -2.2241 | 0.015814 | 1.801 |
| Carnosine | 0.25697 | -1.9603 | 0.024622 | 1.6087 |
| Diethanolamine | 2.7817 | 1.476 | 0.030025 | 1.5225 |
| Methyluridine | 0.38369 | -1.382 | 0.030025 | 1.5225 |
| Aspartate | 2.6075 | 1.3827 | 0.035226 | 1.4531 |
| **4-Fumarylacetoacetate** | 0.49123 | -1.0255 | 0.039728 | 1.4009 |
| **4-Maleylacetoacetate** | 0.49123 | -1.0255 | 0.039728 | 1.4009 |
| Uridine monophosphate (UMP) | 0.45329 | -1.1415 | 0.042379 | 1.3728 |

Supplementary Table 2: Fold change (FC) and adjusted p-values (false discovery rate (FDR) from Benjamini-Hochberg (BH) method) for 34 significantly differential metabolites between EVD survivors with PES and asymptomatic survivors. Metabolites with shared analytical features are bolded and grouped together with the same color font.

| **Variable** | **by Variable** | **Spearman ρ** | **Prob>\|ρ\|** | **BH.corr.pvalue** |
| --- | --- | --- | --- | --- |
| Succinate | Glucose | -0.5960 | 0.0001 | 0.00065941 |
| Methylmalonate | Glucose | -0.5960 | 0.0001 | 0.00065941 |
| Methylmalonate | Succinate | 1 | 0.0001 | 0.00065941 |
| Succinate | Malate | 0.4407 | 0.0063 | 0.02370508 |
| Methylmalonate | Malate | 0.4407 | 0.0063 | 0.02370508 |
| Glucose | Malate | -0.4066 | 0.0125 | 0.04141791 |
| Threonate-1,4-lactone | Glucose | -0.5960 | 0.0001 | 0.00065941 |
| Threonate-1,4-lactone | Succinate | 1 | 0.0001 | 0.00065941 |
| Threonate-1,4-lactone | Methylmalonate | 1 | 0.0001 | 0.00065941 |
| Acrylate | Glucose | 0.9381 | 0.0001 | 0.00065941 |
| Deoxycytidine | Malate | 0.5861 | 0.0001 | 0.00065941 |
| N-Acetylornithine | Glucose | -0.6449 | 0.0001 | 0.00065941 |
| Inosine-5'-monophosphate (IMP) | Glucose | -0.6361 | 0.0001 | 0.00065941 |
| Propanoate | Succinate | 0.826 | 0.0001 | 0.00065941 |
| Propanoate | Methylmalonate | 0.826 | 0.0001 | 0.00065941 |
| 2-Oxo-4-hydroxy-4-carboxy-5-ureidoimidazoline | Glucose | 0.7892 | 0.0001 | 0.00065941 |
| 3-Hydroxy-3-methylglutarate | Glucose | 0.8888 | 0.0001 | 0.00065941 |
| Acetate | Glucose | 0.9263 | 0.0001 | 0.00065941 |
| Acetate | Succinate | -0.5846 | 0.0001 | 0.00065941 |
| Acetate | Methylmalonate | -0.5846 | 0.0001 | 0.00065941 |
| 6-Phospho-gluconate | Succinate | 0.6264 | 0.0001 | 0.00065941 |
| 6-Phospho-gluconate | Methylmalonate | 0.6264 | 0.0001 | 0.00065941 |
| Acrylate | Succinate | -0.5799 | 0.0002 | 0.00122202 |
| Acrylate | Methylmalonate | -0.5799 | 0.0002 | 0.00122202 |
| 2-Oxo-4-hydroxy-4-carboxy-5-ureidoimidazoline | Succinate | -0.5778 | 0.0002 | 0.00122202 |
| 2-Oxo-4-hydroxy-4-carboxy-5-ureidoimidazoline | Methylmalonate | -0.5778 | 0.0002 | 0.00122202 |
| N-Acetylornithine | Succinate | 0.5614 | 0.0003 | 0.00175263 |
| N-Acetylornithine | Methylmalonate | 0.5614 | 0.0003 | 0.00175263 |
| 3-Hydroxy-3-methylglutarate | Succinate | -0.5372 | 0.0006 | 0.00327541 |
| 3-Hydroxy-3-methylglutarate | Methylmalonate | -0.5372 | 0.0006 | 0.00327541 |
| 2-Hydroxyhippurate | Succinate | 0.5332 | 0.0007 | 0.00335396 |
| 2-Hydroxyhippurate | Methylmalonate | 0.5332 | 0.0007 | 0.00335396 |
| Dopaquinone | Succinate | 0.5332 | 0.0007 | 0.00335396 |
| Dopaquinone | Methylmalonate | 0.5332 | 0.0007 | 0.00335396 |
| Leucodopachrome | Succinate | 0.5332 | 0.0007 | 0.00335396 |
| Leucodopachrome | Methylmalonate | 0.5332 | 0.0007 | 0.00335396 |
| alpha-Hydroxyhippurate | Succinate | 0.5332 | 0.0007 | 0.00335396 |
| alpha-Hydroxyhippurate | Methylmalonate | 0.5332 | 0.0007 | 0.00335396 |
| 4- Carboxyphenylglycine | Succinate | 0.5332 | 0.0007 | 0.00335396 |
| 4- Carboxyphenylglycine | Methylmalonate | 0.5332 | 0.0007 | 0.00335396 |
| 6-Phospho-gluconate | Glucose | -0.4844 | 0.0024 | 0.01031226 |
| 2-Oxo-4-hydroxy-4-carboxy-5-ureidoimidazoline | Malate | -0.4704 | 0.0033 | 0.01382264 |
| Deoxycytidine | Glucose | -0.4535 | 0.0048 | 0.01891598 |
| Uridine monophosphate (UMP) | Glucose | -0.4538 | 0.0048 | 0.01891598 |
| Threonate-1,4-lactone | Malate | 0.4407 | 0.0063 | 0.02370508 |
| 6-Phospho-gluconate | 2-Oxoglutarate | -0.4391 | 0.0066 | 0.02455642 |
| Cysteinylglycine | Malate | 0.4365 | 0.0069 | 0.02538895 |
| 3-Hydroxy-3-methylglutarate | Malate | -0.4367 | 0.0069 | 0.02538895 |
| Hypoxanthine | Glucose | -0.4334 | 0.0074 | 0.02693115 |
| 3-Hydroxy-3-methylglutarate | Oxalosuccinate | 0.4294 | 0.008 | 0.02895652 |
| Acrylate | Malate | -0.4286 | 0.0081 | 0.02900323 |
| 2-Oxo-4-hydroxy-4-carboxy-5-ureidoimidazoline | Oxalosuccinate | 0.4229 | 0.0091 | 0.03156563 |
| Acetate | Malate | -0.4229 | 0.0091 | 0.03156563 |
| Acrylate | Oxalosuccinate | 0.408 | 0.0122 | 0.040626 |
| Acetate | Oxalosuccinate | 0.3988 | 0.0145 | 0.04710732 |
| Glucose | Diethanolamine | 0.674 | 0.0001 | 0.00065941 |
| Succinate | gamma-Glutamyl-2-aminobutyrate | 0.5863 | 0.0001 | 0.00065941 |
| Methylmalonate | gamma-Glutamyl-2-aminobutyrate | 0.5863 | 0.0001 | 0.00065941 |
| 2-Oxoglutarate | Ascorbate | 0.811 | 0.0001 | 0.00065941 |
| 2-Oxoglutarate | 4-Fumarylacetoacetate | 0.7044 | 0.0001 | 0.00065941 |
| 2-Oxoglutarate | 4-Maleylacetoacetate | 0.7044 | 0.0001 | 0.00065941 |
| Malate | Ascorbate | 0.5538 | 0.0004 | 0.00225763 |
| Glucose | gamma-Glutamyl-2-aminobutyrate | -0.5555 | 0.0004 | 0.00225763 |
| 2-Oxoglutarate | Carnosine | -0.5344 | 0.0007 | 0.00335396 |
| Succinate | Carnosine | 0.4919 | 0.002 | 0.00888 |
| Methylmalonate | Carnosine | 0.4919 | 0.002 | 0.00888 |
| Succinate | Diethanolamine | -0.4689 | 0.0034 | 0.01389202 |
| Methylmalonate | Diethanolamine | -0.4689 | 0.0034 | 0.01389202 |
| Glucose | Carnosine | -0.4244 | 0.0089 | 0.0313619 |

Supplementary Table 3: Spearman ρ values for metabolites related to TCA cycle or glucose. Metabolites considered related to the TCA cycle and that had significant interactions included succinate, malate, methylmalonate, oxalosuccinate, 2-oxoglutarate. Only correlations with a BH corrected p value of <.05 were included.

| **Component 1** | | |
| --- | --- | --- |
| **Metabolite** | **VIP Score** | **Significance - PES** |
| Acetate | 2.6838 | Upregulated |
| Acrylate | 2.6663 | Upregulated |
| Glucose | 2.6593 | Upregulated |
| 3-Hydroxy-3-methylglutarate | 2.6552 | Upregulated |
| Malate | 2.5089 | Downregulated |
| 2-Oxo-4-hydroxy-4-carboxy-5-ureidoimidazoline | 2.4658 | Upregulated |
| Succinate | 2.4551 | Downregulated |
| Methylmalonate | 2.4551 | Downregulated |
| Threonate-1,4-lactone | 2.4551 | Downregulated |
| Propanoate | 2.3163 | Downregulated |
| LysoPA(16:0/0:0) | 2.2934 | Not significant |
| Lyxose | 2.1664 | Not significant |
| Oxalosuccinate | 2.1586 | Upregulated |
| Tyramine | 2.1061 | Not significant |
| Phenylethanolamine | 2.1061 | Not significant |
| **Component** **2** | | |
| **Metabolite** | **VIP Score** | **Significance - PES** |
| 4-Hydroxy-enol-phenylpyruvate | 2.3796 | Not significant |
| 4-Hydroxyphenylpyruvate | 2.3796 | Not significant |
| Caffeate | 2.3796 | Not significant |
| Acetate | 2.3736 | Upregulated |
| Acrylate | 2.3626 | Upregulated |
| Glucose | 2.3488 | Upregulated |
| 3-Hydroxy-3-methylglutarate | 2.3391 | Upregulated |
| Malate | 2.2013 | Downregulated |
| Succinate | 2.1881 | Downregulated |
| Methylmalonate | 2.1881 | Downregulated |
| Threonate-1,4-lactone | 2.1881 | Downregulated |
| 2-Oxo-4-hydroxy-4-carboxy-5-ureidoimidazoline | 2.1641 | Upregulated |
| Palmitoylcarnitine | 2.125 | Downregulated |
| LysoPA(16:0/0:0) | 2.1045 | Not significant |
| Propanoate | 2.0713 | Downregulated |

Supplementary Table 4: Top 15 variables of importance (VIP) for PLS-DA model. Metabolite considered significantly upregulated or downregulated if FC > 2.0 and FDR <.05 when comparing PES and asymptomatic survivor groups.

**Supplementary Figures**

Supplementary Figure 1: Study Design Focusing on EVD Survivors with PES Compared to Asymptomatic Survivors

The primary analysis compared EVD survivors with PES to asymptomatic survivors. Secondary analyses included comparing EVD survivors with PES and household contacts as well as survivors with MSK/GI sequelae to survivors with cardiopulmonary sequelae. Created in BioRender. “Sanford, A. (2026) https://BioRender.com/w7fms3f“

Supplementary Figure 2: Principal Components Analysis (PCA) Demonstrates Separation of Survivors with PES and Asymptomatic Survivors

EVD survivors with PES separate from asymptomatic survivors based on metabolic profile. Data analyzed in MetaboAnalyst and visualized in JMP.

Supplementary Figure 3: Discovery Models of Biomarkers of PES

Biomarker analysis was performed to identify group membership (PES vs asymptomatic survivor) in MetaboAnalyst. Discovery (n= 39) and validation (n= 18) cohorts were used (discovery cohorts shown here, validation cohorts in Figures 5A-B). Potential biomarkers were selected from PLS-DA and Random Forest (RF) modeling in the discovery cohort. The top 10 most important features for predicting PES were the same in RF and PLS-DA modeling. If the metabolite was higher in the PES or asymptomatic group, the box to the right of the figure was marked red, and if that metabolite was lower, the box was marked blue. Figure 2A: ROC from discovery model from PLS-DA and 10 most important features, ranked by importance. Figure 2B: ROC from discovery model from RF and 10 most important features, ranked by importance
